# Cross-talk and mutual shaping between the immune system and the microbiota during an oyster's life

**DOI:** 10.1098/rstb.2023.0065

**Published:** 2024-05-06

**Authors:** Delphine Destoumieux-Garzón, Caroline Montagnani, Luc Dantan, Noémie de San Nicolas, Marie-Agnès Travers, Léo Duperret, Guillaume M. Charrière, Eve Toulza, Guillaume Mitta, Céline Cosseau, Jean-Michel Escoubas

**Affiliations:** ^1^ IHPE, University of Montpellier, CNRS, IFREMER, University of Perpignan Via Domitia,34090 Montpellier, France; ^2^ Ifremer, IRD, ILM, Université de Polynésie Française, UMR EIO, Vairao 98179, French Polynesia

**Keywords:** holobiont, microbiome, immunity, homeostasis, immune priming, ontogeny

## Abstract

The Pacific oyster *Crassostrea gigas* lives in microbe-rich marine coastal systems subjected to rapid environmental changes. It harbours a diversified and fluctuating microbiota that cohabits with immune cells expressing a diversified immune gene repertoire. In the early stages of oyster development, just after fertilization, the microbiota plays a key role in educating the immune system. Exposure to a rich microbial environment at the larval stage leads to an increase in immune competence throughout the life of the oyster, conferring a better protection against pathogenic infections at later juvenile/adult stages. This beneficial effect, which is intergenerational, is associated with epigenetic remodelling. At juvenile stages, the educated immune system participates in the control of the homeostasis. In particular, the microbiota is fine-tuned by oyster antimicrobial peptides acting through specific and synergistic effects. However, this balance is fragile, as illustrated by the Pacific Oyster Mortality Syndrome, a disease causing mass mortalities in oysters worldwide. In this disease, the weakening of oyster immune defences by OsHV-1 µVar virus induces a dysbiosis leading to fatal sepsis. This review illustrates the continuous interaction between the highly diversified oyster immune system and its dynamic microbiota throughout its life, and the importance of this cross-talk for oyster health.

This article is part of the theme issue ‘Sculpting the microbiome: how host factors determine and respond to microbial colonization’.

## 1. Introduction

Over the past decade, major scientific interest has been given to the role of microbiomes in the biology of metazoans. In particular, an abundant literature has been produced on the dialogue between the immune system and microbiota of animal species. Across the animal kingdom, key studies have highlighted the importance of this dialogue for animal health. It is now recognized that effectors of the immune system control the microbiota and maintain homeostasis with resident microbial communities hosted by vertebrates [1], arthropods [2] and cnidarians [3]. Reciprocally, in both vertebrates and arthropods, the immune system is shaped by resident microbes [4,5] with far-reaching effects on host physiology [6,7].

The immunity-microbiota cross-talk and its physiological consequences have remained much less explored in molluscs until recently. Within this phylum, bivalve molluscs (e.g. oysters, mussels, clams) are particularly interesting as they encounter important microbial challenges in their natural habitats (intertidial coastal marine systems, lagoons and estuaries) from the very first embryonic and larval stages, when they are still ciliated and move freely in the seawater column, to the benthic spat and adult stages when they feed on plankton and their associated microorganisms. Benthic oysters not only host microbial communities on their body surfaces [8] but also in their circulating body fluids, i.e. the haemolymph (equivalent of the blood) [9], in the pallial/extrapallial cavity fluid [10,11] as well as in all the tissues and developmental stages studied [12,13]. As they are filter feeders able to concentrate microorganisms present in their environment, it has been argued that bivalves host transient microbial communities that vary with environmental conditions. This is actually observed for a fair proportion of their microbiota, which is rapidly eliminated upon depuration with purified seawater, a common method employed for removing waterborne microbial contaminants from bivalves [14]. Still, the microbiota of bivalves is highly distinct from the microbial communities found in the surrounding seawater [8,9,14–16]. Moreover, among bivalve-associated bacteria, some genera, which share the same habitat and may have a long co-evolutionary history with their hosts, are little affected by environmental variations [14,17]. Still, it should be acknowledged that even when some bacterial taxa are consistently found in bivalves, their classification typically only goes as specific as the genus level. True co-evolution would involve interactions between oysters and distinct strains or populations of bacteria. Moreover, with the exception of bivalve species that host chemosynthetic bacterial mutualists (for review see [18]), such as the lucinid *Codakia orbiculate* hosting sulfur-oxidizing bacteria [19], most of the time it is ignored whether members of the microbiota have key functions in the biology of bivalves.

Oysters are fascinating organisms in which to study the cross-talk between the microbiota and the immune system, and their reciprocal influence. These organisms indeed host highly diverse and dynamic microbiota, which have shaped a vastly expanded and diversified innate immune repertoire [20,21], a diversification that may have evolved to fight pathogens and/or tolerate certain members of the microbiota. Studies on the oyster *Crassostrea gigas* have revealed a tight connection between the microbiota and the oyster health status [15]. Bivalve microbiota may be manipulated to prevent disease in aquaculture [22]. A fine-tuned dialogue between oyster immunity and its microbiota was evidenced from early life to adult stages [23–25]. Recently a first insight into microbial community gene functions has been made accessible, either by shotgun metagenomics [11] or metatranscriptomics [26]. The present article summarizes the knowledge accumulated on oyster–microbiota interactions over the last few years and reviews the dysregulations leading to dysbiosis and oyster death. Finally, it covers the avenues opened for disease prevention and control.

## 2. Homeostasis: a diversified and fluctuating microbiota cohabits with oyster immune cells

**Figure 1.**
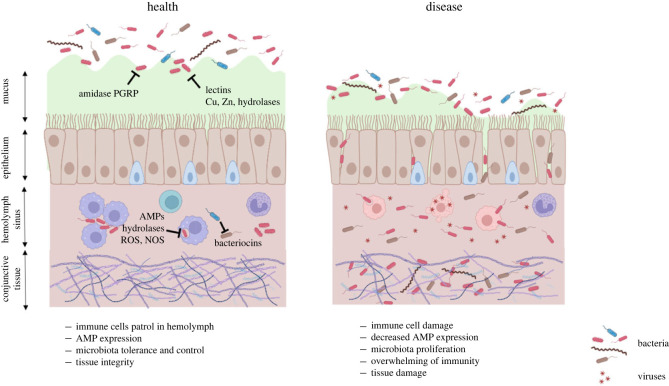
Immune–microbiota interplay at an oyster epithelium: homeostasis versus dysbiosis. The left panel (health) illustrates a homeostatic context. The microbiota is kept away from epithetial cells (brown cells) by a thick mucus layer (green) secreted by mucocytes (blue cells), which covers the oyster body surfaces. Lectins, hydrolases, copper and zinc contribute to limit penetration of microorganisms. It is assumed that amidase peptidoglycan recognition proteins (PGRPs) prevent peptidoglycan from activating immune receptors. Bacteria circulate in the haemolymph (pink) without inducing a measurable immune response. They are kept under control by functional immune cells (purple) and the AMPs, hydrolases, reactive oxygen species (ROS) and nitric oxide synthase (NOS) they produce. Bacteriocins mediate competition. The right panel (disease) shows a context of dysbiosis. Pathogens alter immune cell functions (pink cells), which no longer control the microbiota. AMP expression is altered. Bacteria invade the conjunctive tissues. Tissues lose their integrity and their barrier functions. Figure created using biorender.com.

Oyster larvae, as soon as two days after reproduction, are equipped with a velum used for motility and feeding, and two valves develop to form the shell. Oysters become sessile after 2.5 weeks when a foot develops to attach to a hard surface (pediveliger stage). Once permanently attached, the oyster becomes a spat. From this stage until adulthood, the oyster soft body is covered by the mantle (or pallium), a tissue that ensures the development and growth of the shell and also plays a sensory role. A pair of lamellar gills, which extend from one extremity of the gut to the other, ensures the functions of respiration, nutrition and excretion of certain waste products. A kidney consisting of a tubular gland located within a renal sinus comprises the excretory system. Oysters have a open-type circulatory system: the circulating fluid called haemolymph (equivalent to vertebrate blood) is not confined to the vessels and the heart but flows into sinuses and infiltrates the intercellular space of all tissues (for review see [27]). The haemolymph carries the circulating immune cells called haemocytes throughout the oyster body. The heart propels the haemolymph into the circulatory system. The two oyster valves forming the shell, which protect the oyster body, also enclose the pallial and extrapallial cavity fluids, which surrounds the mantle and the soft body of the mollusc, and contains haemocytes. Thus, oyster body surfaces are covered by the pallial fluid/mucus, which contains high amounts of antimicrobials such as copper, zinc and hydrolases such as lysozyme participating in oyster chemical defenses against infections [28,29]. The mucus is secreted by mucocytes lining epithelia. Together with ciliary movement at epithelial surfaces, the mucus is believed to restrict microbial growth and serves as a first line of defence against infections from environmental pathogens (figure 1). Remarkably, bacteria are barely seen attached to epithelia and mucus of healthy oysters by means of electron microscopy [30]. By contrast, bacteria are easily detected in oyster body fluids [10]. While body fluids may represent an entry route for oyster tissue colonization, they also host a number of bacterial genera such as *Pseudoalteromonas* that may confer protection through the production of antimicrobial compounds. Such protective bacteria have been isolated both in the pallial fluid of *Crassostrea virginica* [31] and in the haemolymph of *C. gigas* [32]*.* They secrete antimicrobial peptides (AMPs), generally referred to as bacteriocins [33]*,* which confer a protective shield against pathogens.

Taxonomic information on the microbiota associated with oyster tissues has been facilitated by increased access to next generation sequencing. The advent of this technology has highlighted the tremendous plasticity of the oyster microbiota under the influence of both biotic (e.g. oyster genetic background, developmental stage and metabolic rate or nutrient availability) and abiotic factors (i.e. temperature, pH, salinity, *p*CO_2_) [34–38]. In addition to important fluctuations in response to environmental changes, physiological status and developmental stages, the oyster microbiota shows important inter-individual variability, both at the level of the whole animal and at the level of individual tissues throughout all life stages [8,11–13,39]. This heterogeneity has made it difficult to identify the core bacterial microbiota in oysters, if one exists. Overall, only Spirochaetaceae were widely distributed across tissues of different oyster species (*C. gigas, C. virginica* and *Saccostrea glomerata*) living on three continents [11,12,15,35,39–41]. Such a trans-specific conservation may be linked to the ecology of spirochaetes, which are abundant in intertidial marine sediments [42] to which oysters are exposed. By sharing the same habitat, spirochaetes may have evolved an oyster-associated life style, but accidental associations cannot be dismissed. At an oyster population scale, a number of rare bacterial species were associated with genetic differentiation of *C. gigas* oysters but this association was lost in stressful environmental conditions [38]. More conclusively, oyster microbiota analyses carried out at the whole animal and tissue levels have shown that each tissue harbours its own microbial consortia. However, it is still unclear whether these communities endow specific functions in the biology of each tissue [8,11,12] and/or if they are simply shaped by the diversity of microhabitats they inhabit.

Microbiota of the haemolymph have been the most extensively studied in oyster health and disease, probably due to the key role of the haemolymph in immunity. Haemolymph bacterial communities can be clearly distinguished from those found in seawater and solid tissues, but the community dynamics show a close relationship to the internal and external oyster environment, suggesting potential host selection [8,9,11,14]. Beyond its bacterial component, oyster haemolymph contains diverse protists and viruses; the composition and dynamics of these communities depend on the environment as well as on oyster genetics [9]. Among the diverse components of the haemolymph microbiota, bacteria show a more stable taxonomic composition than protists and viruses, suggesting more fine-tuned interactions with the oyster host. Based on these results, the authors have suggested that some bacterial groups, mostly γ- and α-proteobacteria, may be well adapted to the haemolymph conditions, whereas viral and protist communities may be primarily transient [9]. This remains to be confirmed since microbiota studies are strongly biased towards the bacterial component and only a very limited number of studies have focused on viruses and protists.

It is far from understood how homeostasis is maintained in the complex ecosystem of the haemolymph. Particularly, it is still largely unknown how such an abundant and diverse microbiota coexists and is tolerated by the host haemocytes [43], which patrol in the haemolymph (figure 1) and are equipped with key immune receptors, pathways and effectors conserved across the animal kingdom [44]. Remarkably, a number of these receptors and effectors have highly diversified in oysters, some of them belonging to large multigene families involved in specific immune responses [21]. Recognition of potential pathogens occurs in the haemolymph where they are contained by phagocytosis and haemocyte aggregation, preventing them from invading the connective tissue [45] (figure 1). To persist in such a hostile environment, the resident microbiota of oysters may have evolved low immunogenic properties, as recently evidenced in a *Vibrio* population showing preferential association with oyster tissues. In this population, a modified O-antigen structure was shown to reduce strain recognition by oyster immune receptors [46], unlike in other *Vibrio* that behave as opportunistic pathogens and colonize oysters by actively altering their cellular defences, thereby favouring immune evasion [45,47]. In addition, the oyster mucus proteins may confer immune tolerance. Indeed a number of secreted amidase PGRPs (peptidoglycan recognition proteins) have been identified in oyster mucus covering pallial organs (gill, mantle, labial palps) [29]. In many species, from insects to mammals, amidase PRGPs are capable of cleaving the bacterial cell-wall glycopeptides released by bacteria into non-immunogenic compounds. Thereby they control the intensity of the immune response and act as detoxifying enzymes [48]. Their secretion in oyster mucus is an important indication that they may participate in the maintenance of homeostasis at the immune-bacteria interface.

## 3. The immune system shapes the microbiota

Cellular defences are highly potent in oysters. Oyster immune cells, the haemocytes, appear during gastrula-trochophore stages and they already express immune genes at the trochophore stage (15 h post fertilisation, hpf), suggesting the initiation of the immune system [49,50]. However, at hatching (9 hpf), oyster larvae tend to be immune-depleted, which makes them more susceptible to infections. From the D-veliger larval stage (17 hpf), haemocytes perform phagocytosis [49]. In spat (greater than 2.5 weeks post fertilisation) and adults, haemocytes circulate in haemolymph and infiltrate injured/infected tissues. They not only perform efficient phagocytosis but they produce key antimicrobials (reactive oxygen and nitrogen species, antimicrobial peptides and proteins, hydrolases) capable of killing microbes both intracellularly and extracellularly (for review see [51]) (figure 1, left panel). The potent respiratory burst produced by haemocytes triggers a mechanism of ETosis by which haemocytes release DNA Extracellular Traps that contain antimicrobial histones and entrap bacteria, preventing them from disseminating outside the haemolymph or sites of injury [52]. Most oyster bacterial pathogens have evolved the capacity to escape oyster cellular defences, either by evading phagocytosis [53] or by exerting cytotoxicity against haemocytes [45,47], thereby causing systemic infections (figure 1, right panel). Such mechanisms of cytotoxicity are rarely observed in non-pathogenic bacteria [54], which instead are contained in the circulation by haemocyte clumps [45]. This provides indirect evidence that haemocytes participate in keeping the commensal microbiota under control.

Antimicrobial peptides (AMPs) have been shown to combat infections in a broad number of species across the animal kingdom including marine invertebrates [55]. However, it is only in recent years that AMPs have been shown to play a key role in controlling microbiota hosted by metazoan hosts. The main families of antimicrobial peptides and proteins identified in oysters include CS*αβ* defensins called *Cg-*Defs in *C. gigas* oysters (highly conserved in fungi, plants, arthropods, molluscs, but they were lost in chordates), bactericidal/permeability increasing proteins (*Cg*-BPI; found both in vertebrates and molluscs), and big defensins (*Cg-*BigDefs; found in cephalochordates, chelicerates, molluscs and other lophotrochozoans). *Cg-*Defs and *Cg-*BPI are expressed by haemocytes and epithelial cells, contributing both to local and systemic responses (for review see [43]). *Cg-*BigDefs are expressed by oyster haemocytes only [56]. Although transcripts of *Cg-*Defs have been found in oyster embryos, their expression increases significantly in the first larval stages, from hatching to the D-veliger stage [49]. This indicates that AMPs may play a role at the immune-microbiota interface early during development. Oyster antimicrobial peptides and proteins have very distinct and complementary mechanisms of action, which enables them to target a broad diversity of microorganisms. *Cg-*Defs are ligands of lipid II that inhibit peptidoglycan synthesis; they are essentially active against Gram-positive bacteria [57]. *Cg-*BPI, which efficiently binds lipopolysaccharide, damages membranes of Gram-negative bacteria [58]. *Cg-*BigDefs, which autoassemble and entrap bacteria in supramolecular structures called nanonets, have broad spectrum antimicrobial activities [59]. Unlike in other species of molluscs (e.g. mussels, [60]), AMPs in oysters are not expressed at high concentrations. Instead, most AMP families have expanded and diversified through gene duplication events followed by rapid molecular diversification [56], as a result of directional selection pressures [61]. This molecular diversity creates synergy and enlarges the spectrum of antimicrobial activities of oyster AMP families, particularly *Cg-*Defs and *Cg-*BigDefs [24,62]. Remarkably, the gene expansion and sequence diversification of *Cg-*BigDefs has also conferred specificity to members of this peptide family against bacteria belonging to the oyster microbiota [24]. Unlike in the scallop *Argopecten purpuratus*, where only one big defensin gene has strong effects on the composition of the microbiota [63], *Cg-*BigDefs in oysters fine-tune the microbiota, probably through their very specific activities against different members of the microbiota [24]. Such a specificity has been recently discovered in AMPs from other animal phyla, e.g. in insects [64]. It was recently proposed that individual diptericins in *Drosophila* have been selected to control specific harmful bacteria present in their microbiome [65]. Until now we ignore whether variation in presence/absence of *Cg-*BigDefs in oysters [66] has been shaped to fight pathogens and/or to tolerate beneficial microbes.

While lectins have remained much less explored for their interactions with the oyster microbiota, they are highly diverse in oysters and abundant in the mucus covering oyster epithelial surfaces [29]. Lectins found in oyster mucus have been attributed to infiltrating haemocytes, mucocytes and epithelial cells. This attribution is based on a comparison of proteins found in mucosal secretions with proteomic and transcriptomic data from specific oyster tissues [29]. Among their diverse functions, lectins are involved in host–microbe interactions both in parasitic and mutualistic contexts. For instance, C-type lectins in shrimp agglutinate bacteria selectively [67] and they maintain the homeostasis of the intestinal and haemolymph microbiota [68,69]. In oysters, C-type lectins may participate in the control of bacteria as demonstrated for *Cg*CLec-3, which is expressed by haemocytes and agglutinates specific bacterial species [70]. The massive diversification of C-type lectins in the genome of *C. gigas* (154 genes) [20] indicates an uncovered diversity of putative functions and putative specificities in the interaction with the microbiota. A similar phenomenon of diversification was decribed for the complement C1q domain containing proteins (164 genes in *C. gigas*), whose expression is induced upon biotic challenge [21], and which may act as opsonins to promote phagocytosis as shown for the plasma protein p1-*Cg*C1q [71].

A number of pallial mucus proteins likely to recognize and/or control parasites and microbes have been identified [29]. These include several invertebrate thioester-containing proteins (TEPs) similar to *Biomphalaria glabrata* *Bg*TEP, which forms an immune complex with fibrinogen-related proteins produced by the molluscan host and mucins produced by the parasite *Schistosoma mansoni* [72]*.* This haemocyte-expressed protein binds to a diversity of bacteria suggesting an opsonin role [73], like in insects [74,75]. Pallial mucus also contains the DMBT-1 protein (deleted in malignant brain tumors 1 protein) [29], which functions in mucosal immunity by mediating bacterial recognition and countering invasion [76]. In *C. gigas* its expression is suppressed by pathogenic *Vibrio*, which have the ability to bypass host defences [45]. Finally, two ferritins were found in the pallial mucus [29]. Ferritins are iron-scavenging proteins that participate in the so-called nutritional immunity. By depriving bacteria of iron, these host proteins can help limit bacterial growth. The fight for iron is key in the control of infections by *Vibrio*, which produce their own siderophores, such as vibrioferrin, for the uptake of this essential nutrient [77]. Recent results have shown the key role of iron homeostasis in the structuring and assembly of pathological microbial communities in oysters [47].

## 4. The microbiota shapes the oyster immune system

Multicellular organisms exist as complex ecosystems, also known as holobionts, composed of both the animal host and its associated microbiota [78]. Interactions between the host and its microbiota implies complex feedbacks. If the immune system orchestrates the maintenance of key features of the host–microbiota interactions and establishment of the microbiota community, the latter, in return, plays a critical role in the development, education and function of the immune system [5,79,80]. The sequencing of the *C. gigas* genome has revealed a remarkable expansion and functional divergence of immune gene families in this species [20,21]. Their expression profiles revealed specific responses to a diversity of microbial challenges. On an evolutionary scale, it has been argued that the dynamic and pathogen-rich environment of oysters has created complex biotic and abiotic stresses, constituting strong selection pressures that have led to a major diversification of the immune system. On the scale of a lifetime, the early life is recognized as a crucial window of opportunity during which microbial colonization sets appropriate immune development and establishes the foundation for lifelong immunity. Studies in humans and in a vast array of vertebrates or invertebrate animals has demonstrated that colonization of complex microbiota or single symbionts in early life does not only impact the maturation of the mucosal and gut immune systems, but also modulates the systemic immune response [81–87]. In addition, there is compelling evidence that early life environments can induce long-lasting changes in the immune system of progenies and have critical impacts on health and disease. Notably, it has been shown that nonpathogenic microbial exposures during critical periods of development in mammals favourably imprint immunity, promoting protective immunity that reduces risk of disease later in life [88].

**Figure 2.**
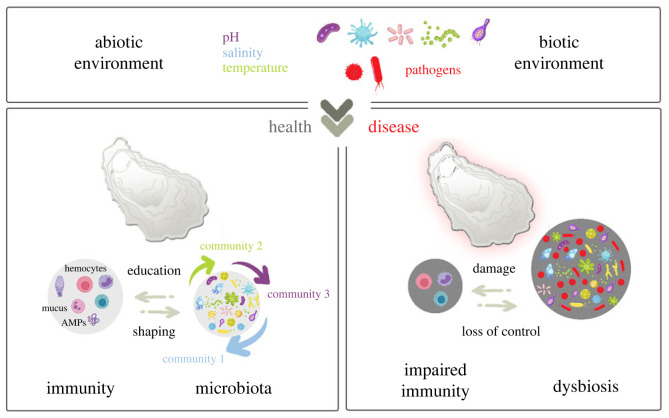
Environmental factors acting on the cross-talk between the oyster immune sytem and its microbiota. Healthy microbiota is diverse and plastic (left panel). Its structure is influenced by environmental factors; for instance, some communities can be favoured by given abiotic factors (pH, temperature or salinity). Microbiota plays a key role in the education of the immune system. In parallel, the immune system relying on haemocytes, mucus production and secreted molecular effectors (e.g. AMPs) participates in the control of the homeostasis of the oyster microbiota. In the presence of pathogens, which can alter immune defences, loss of control of oyster microbiota results in dysbiosis and leads to fatal sepsis (right panel).

As filter feeders, oysters evolve in a rich microbial environment, under constant interaction with pathogenic, commensal and beneficial microorganisms. In a recent study, Unzueta-Martínez *et al.* showed that not all members of the oyster-associated microbiota are governed by the same ecological dynamics and that both horizontal and vertical transmission routes are possible [13]. Particularly the authors identified some members of the bacterial communities that establish at early developmental stages (from gametes to spats) and persist across multiple life stages [13]. Since initiation of the immune system occurs early in the oyster development [49,50], this early microbiota association raises numerous questions about its impact on the immune system maturation. In *C. gigas*, another recent study showed that an early larval exposure to a non-infectious environmental microbiota can induce a systemic immune response that confers a protection against the Pacific Oyster Mortality Syndrome [25]. Remarkably, this early exposure resulted in a lifelong and intergenerational increased immune competency that persisted far beyond the initial exposure. The exposure impacted the diversity and shifted the composition of the oyster microbiota. Because of the overall versatility of oyster microbiota, we can speculate that this control over microbiota takes place at the functional group level by shaping a community of bacteria with various taxonomies that perform key functions. Increased immune competency was supported by a long-term reprogramming of immune gene expression within and across generations, and notably correlated with differential expression of conserved pathogen recognition receptors (PGRP, lectins, scavenger receptors, TLR, RLR, macrophage receptor), innate immune pathways (IFN-TLR-JAK/STAT pathways) and antimicrobial effectors (TNF, proteinases, SOD, interferon-stimulated genes, AMPs). Moreover, this phenotype was associated with heritable changes in epigenetic signatures (DNA methylation patterns) that are reminiscent of mechanisms underlying innate immune memory response in mammals and plants [89,90]. Remarkably, epigenetic variations affecting immune pathways have been shown to confer rapid adaptation to pathogenic pressure in oyster [91]. The hypothesis beyond a microbiota-induced memory is a continuous reshaping of cellular signaling pathways and microbiota, which resulted in long-term heritable epigenetic imprinting (figure 2, left panel). The study by Fallet *et al*. [25] thus perfectly illustrates the continuous cross-talk between host and microbiota and its role in disease susceptibility or resilience.

This study resonates with emerging concepts on the impact of environmental signals, either biotic or abiotic, on epigenetic changes responsible for heritable phenotypic outcomes [92–94] and on innate immune memory formation (also known as trained immunity) [89,90,95]. In mammals and arthropods, commensal microbiota was shown to shape immune capacities, not only at early stages, and to have a systemic effect on the immune response, inducing enhanced resistance towards a vast array of unrelated pathogens [96–103]. These findings are reminiscent of evidence of symbiont-mediated immune priming (reviewed in [104]) showing the impact of beneficial symbionts on immune capacities. In *C. gigas*, long-term immune priming capacities were evidenced using either non-pathogenic bacteria or viral mimic pre-conditioning [105,106]. These data confirmed that non-pathogenic microbial exposures can promote the generation of protective immunity, further suggesting complex interactions between microbiota and immunity, and the potential for some members of oyster microbiota to drive innate immune memory and support enhanced survival.

## 5. Fatal breakdown of homeostasis: loss of control of the microbiota

Homeostasis is crucial to maintain internal stability and balance in the face of fluctuating environmental conditions. Studies on cnidarians nicely illustrate that the stability of the association between a host and its microbiota is crucial for health [107] and to cope with the environment [108,109]. In humans, dysbiosis leads to disease development and progression, through dysregulation of community composition, modulation of host immune response, and induction of chronic inflammation [110].

As oysters are both osmoconformers and eurythermal, the microbial communities they host are naturally exposed to significant changes in osmolarity and temperature over days and seasons, two factors that modulate microbial community dynamics, either directly or indirectly, by modulating oyster physiology and immunity [111]. Not surprisingly, healthy oysters—which exhibit extended physiological limits—also tolerate important variations in the structure of their microbiota (see §2 and figure 2, left panel). At the metaorganism level (the holobiont), this plasticity may increase oyster adaptatibility to rapidly changing environmental conditions, which they experience in intertidial coastal marine systems. However, the balance between a fluctuating microbiota and an effective—but still tolerant—immune system is fragile. Like in other animal species, a compromised homeostasis can have detrimental effects on oyster health by creating an unfavourable environment for the beneficial microbial populations and promoting the often irreversible proliferation of harmful microorganisms. Thus, in some instances temperature fluctuations, changes in salinity, acidification, hypoxia, antibiotic exposure or nutrient availability alter oyster physiology in a way that it affects their ability to maintain a stable internal environment [112,113]. Thereby, environmental factors can contribute to the breakdown of homeostasis (figure 2, right panel). Thus, the highly dynamic composition of oyster haemolymph microbiota can be indicative of major changes in oyster health status. It is actually often accompanied by drastic changes in the composition of the haemocyte formula (i.e. the relative abundance of the different populations of haemocytes) and counts, and a rapid migration of haemocytes in oyster tissues (for review see [51]). For instance, heat stress decreases the stability of the haemolymph microbiota in *C. gigas*, resulting in increased mortality in response to infections [114]. Particularly, destabilization of the oyster haemolymph microbiota can facilitate infection by *Vibrio* [40], which are recruited from the oyster environment [47]. Dramatic events are observed when an abiotic stress co-occurs with an exposure to a pathogen. For instance, marine heatwaves*,* i.e. ‘discrete prolonged anomalously warm water events’, enable proliferation of *Vibrio* in oyster tissues and lead to mass mortality events [115]. Similarly, shifts in temperature control the fatal outcome of *V. aesturianus* infections in oyster [116]. In this particular case, the bacterial microbiota shifts from highly diversified to dominated by one single species [117,118].

Interactions between the immune system and microbiota have strong effects on development of Pacific Oyster Mortality Syndrome (POMS), a major threat to the aquaculture industry. POMS, which is both polymicrobial and multifactorial [119,120], is a classic example of biotic and abiotic factors triggering dysbiosis and leading to oyster death. When the seawater temperature rises above 16°C, important changes in the microbiota occur in response to infection with the OsHV-1 µVar virus: the bacterial groups which are normally the most abundant tend to decrease while some rare and opportunistic bacteria proliferate and become pathobionts, particularly *Vibrio* [112] and *Arcobacter* [117]. Elucidating the sequence of events involved in POMS development by comparing resistant and susceptible oyster families revealed that microbiota homeostasis breakdown is central to the pathogenesis process [23]. Sequentially, the infection by OsHV-1 µVar triggers an immune-compromised state that induces microbiota dysbiosis and subsequent bacteraemia, ultimately leading to oyster death. Fatal dysbiosis is accompanied by invasion of the connective tissue by opportunistic bacteria and in particular *Vibrio* and *Arcobacter*, which are consistently associated with oyster death [26]. The virus alters antibacterial defences by infecting haemocytes and reprogramming their functions. OsHV-1 µVar alters more particularly AMP expression, which coincides with a loss of control of microbial communities 24 h after viral infection [23]. The sequence of events leading to dysbiosis and oyster death is conserved across infectious environments in France (Mediterreanean, Atlantic) and oyster genetic backgrounds [26]. Moreover, using a combination of amplicon sequencing and metatranscriptomics, a core pathobiota assemblage was identified; it colonizes oysters during the secondary bacterial infection of oysters in POMS. *Arcobacter*, *Vibrio*, but also *Amphritea*, *Marinobacterium*, *Marinomonas*, *Oceanospirillum* and *Pseudoalteromonas*, together represented up to 40% of the bacterial gene expression at the onset of oyster mortality. Bacteria of the *Vibrio* genus associated to POMS (*V. crassostrea, V. tasmaniensis* or *V. harveyi* according to environments) were shown to actively participate in the pathogenic process by dampening oyster cellular defences and by producing public goods such as siderophores, which benefit not only the producer but also other members of the population or local community to import iron, which is key for bacterial growth [45,47]. Such cooperative behaviours, which make oyster a more favourable environment for microbial proliferation, were key in structuring the *Vibrio* community associated with POMS [47]. Much more remains to be explored regarding the social interactions at play (cooperation, cheating, competition) in the POMS pathobiota. The transcriptional response of the pathobiota was conserved between Mediterranean and Antlantic environments with a major increase in general metabolic pathways reflecting a highly active microbial community growing on amino acids and lipids from dying oyster tissues. Members of the pathobiota overexpressed different metabolic pathways, reflecting differential use of the nutritive resources provided by the diseased oyster host. The complementarity observed could be due to the synergy between various genera participating in different stages of biogeochemical cycles, with the growth of one genus promoting the growth of others. It may also be attributed to limited competition for resources among these genera, implying an efficient utilization of the diverse resources in the oyster environment, supporting the robust growth of bacteria with distinct metabolic characteristics. This functional complementarity may explain the taxonomic conservation of the POMS pathobiota [26].

## 6. Implications for applied perspectives

### (a) Probiotics

A probiotic is, by the definition of the Food and Agriculture Organization (FAO), a ‘live microorganism, which when consumed in adequate amounts, confers a health benefit on the host’ [121]. The use of probiotic strains is already developed for animal production, and it is considered a promising eco-responsible and prophylactic alternative to antibiotics [122]. Probiotics can compete with pathogens by producing diverse antimicrobial substances (bacteriocins, antioxidant molecules), modulating the innate immune system of the host, interfering with microbial communication systems (quorum quenching effect), producing beneficial metabolites, and helping for nutrient adsorption [123]. Regarding probiotics for oyster farming, two of the above strategies are being developed: the direct competition with pathogens and the modulation of the innate immune system [124]. For example, exposure of *C. gigas* larvae to *Pseudoalteromonas* sp. capable of inhibiting the growth of *V. coralliilyticus* improved larval survival during *V. coralliilyticus* infection [125]. Similarly, the administration of *Streptomyces* sp. strains RL8 to juvenile *Crassostrea sikamea* oysters induced significantly higher weight gain and increased antioxidant activity [126].

The natural microbiota of oysters may help identify new probiotics candidates to be used in prophylactic measures. Because of the versatility of oyster microbiota and the rapid disturbance of host genotype–microbial community associations upon external stress, it has been argued that microbiota may be difficult to manipulate and use to improve oyster resistance to disease [38]. It may indeed be difficult to implant probiotic strains stably in oysters. Nevertheless, oyster families with contrasting susceptibility to POMS harbour different microbiota compositions [9,15,112]. Bacterial taxa Colwelliaceae, Cyanobacteria and Rhodobacteraceae are indeed significantly associated with oyster families harbouring higher resistance to POMS [15]. Beyond members of the oyster microbiota, cohabitation of oysters with macroalgae mitigates their resistance to POMS by modifying microbiota composition and affecting transcriptional response to OsHV-1 infection [127]. We argue that modulating oyster physiology and immune system through microbial exposure is a promising way to increase resistance to pathogens.

### (b) Microbial education and immune priming

Another promising use of natural microorganisms as a tool to fight infectious diseases indeed consists in educating the host immune system during its ontogenesis. It is expected that focusing on this crucial developmental stage will result in lifelong and potentially transgenerational beneficial immune properties. Such a microbial education has already been applied successfully in fish [83] and molluscs [25]. More studies are still needed for understanding the basic mechanisms of early life oyster–microbiome interactions, the bases of the immune system developmental plasticity in oysters, and how this may be translated into applications. Already, microbial management methods are applied in aquaculture to promote healthy microbe–larvae interactions [128–131]. The immunomodulatory properties and long-term health benefits of such practices have been demonstrated in fish and shrimp [132]. Further studies are necessary to apply such strategies during oyster larval rearing. This will require further exploration on how the cross-talk between oyster and microbiota is established during development, with emphasis on critical temporal windows. It will also be necessary to precisely define the contributions of specific microbial species of oyster origin to early oyster life immune imprinting and to oyster health status at a juvenile/adult stage. Recent study on the impact of microbiota on oyster immune capacities helped identify bacterial taxa and species overrepresented in animals exposed to microbiota at an early developemental stage, which show increased immune capacities (Rhodobacteraceae, Halomonadaceae, Shewanellaceae and Oceanospirillaceae) [25]. This could help design microbiome-based prophylactic strategies and/or disease mitigation strategies with microbiota species acting as modulators of immune responses (essentially applicable in hatcheries, at stages were larvae can be kept in recirculated water). Elucidating the exact molecular relationship between microbe-derived metabolites, host immune signalling pathways, epigenetic modifications and host physiology will need further investigation.

In addition, a growing body of evidence shows that the innate immune system displays memory traits providing a survival advantage within and across generations [133]. Immune plasticity and memory capacities have been evidenced in molluscs including oysters [25,105,134,135]. Building on these properties could help design pseudo-vaccination strategies (known as immune-priming) based on nonpathogenic microbial exposures to promote protective immunity in individuals and their offspring.

## 7. Conclusion and future directions

Recent literature has highlighted the highly dynamic nature of the oyster bacterial microbiota, under the influence of a rapidly fluctuating biotic and abiotic environment and host factors (genetics, development and physiology). Such dynamic microbiota may have contributed to shaping a highly diversified repertoire of oyster immune genes [20] whose functions remain far from being understood. This makes the oyster a fascinating organism to investigate the cross-talk and reciprocal shaping of the microbiota and the immune system. While microbial communities were shown to educate the immune system during the early stages of oyster development, the role they play at an adult stage remains unclear. Answering such an essential question requires a deeper understanding of microbial community gene functions, beyond descriptions of their taxonomic composition. Although recent progress has been made in this direction [11,26,136], it is not clear yet whether oyster microbiota is shaped according to microbial community gene function and regulation, particularly in a healthy context. To address this challenging question, several difficulties have to be circumvented. First, in order to reveal the array of functions undertaken by microbial communities within distinct tissue environments, it is imperative to examine the biogeography of microbiota gene expression at more detailed levels. Second, beyond bacterial communities of the oyster that are typically studied, it is essential to consider diverse microbial members (e.g. protists, viruses) to fully understand the roles of microbiota in health and disease. Indeed, oyster-associated bacteriophages have co-evolved with *Vibrio* populations causing pathologies in oysters [137]. Furthermore, bacteriophages play a key role in the marine environment by modifying the metabolism of infected bacteria by altering the expression of auxiliary metabolic genes and reorienting host gene expression patterns [138]. This highlights the need to consider the complexity of the microbiota if we are to fully understand its role in oyster health and disease. Future studies should also address the role and specificities of host factors in shaping oyster-associated microbial communities. Indeed, not only is there often a strong genetic component in the capacity of oysters to control infections [139], but there are also key physiological determinants controlled by the environment (e.g. oyster reproduction stage) or the developmental stage that need further investigation [119]. Finally, many immune factors remain poorly characterised in oysters. Despite their key role in controlling infections, the diversity of oyster haemocytes (in terms of lineage, gene expression, activity) and their interactions with commensal and beneficial microbes have received little study. Similarly, oysters are known to have impressive immune gene and AMP diversification, which may have evolved to adapt to changing microbial environments, as recently evidenced for some insect AMPs [65]. However, the roles of many of these diversified oyster immune genes in the fight against pathogens and the tolerance of beneficial microbial communities are as yet unknown.

## Data Availability

This article has no additional data.

## References

[1] Bevins CL, Salzman NH. 2011 Paneth cells, antimicrobial peptides and maintenance of intestinal homeostasis. Nat. Rev. Microbiol. **9**, 356-368. (10.1038/nrmicro2546) 21423246

[2] Marra A, Hanson MA, Kondo S, Erkosar B, Lemaitre B. 2021 *Drosophila* antimicrobial peptides and lysozymes regulate gut microbiota composition and abundance. mBio **12**, e00824-21. (10.1128/mBio.00824-21) 34253067 PMC8406169

[3] Franzenburg S, Walter J, Künzel S, Wang J, Baines JF, Bosch TCG, Fraune S. 2013 Distinct antimicrobial peptide expression determines host species-specific bacterial associations. Proc. Natl Acad. Sci. USA **110**, E3730-E3738. (10.1073/pnas.1304960110) 24003149 PMC3785777

[4] Broderick NA, Buchon N, Lemaitre B. 2014 Microbiota-induced changes in *Drosophila melanogaster* host gene expression and gut morphology. mBio **5**, e01117-e01114. (10.1128/mBio.01117-14) PMC4045073 24865556

[5] Hooper LV, Littman DR, Macpherson AJ. 2012 Interactions between the microbiota and the immune system. Science **336**, 1268-1273. (10.1126/science.1223490) 22674334 PMC4420145

[6] Liu X, Nagy P, Bonfini A, Houtz P, Bing X-L, Yang X, Buchon N. 2022 Microbes affect gut epithelial cell composition through immune-dependent regulation of intestinal stem cell differentiation. Cell Reports **38**, 110572. (10.1016/j.celrep.2022.110572) 35354023 PMC9078081

[7] Miani M et al. 2018 Gut microbiota-stimulated innate lymphoid cells support β-defensin 14 expression in pancreatic endocrine cells, preventing autoimmune diabetes. Cell Metab. **28**, 557-572. (10.1016/j.cmet.2018.06.012) 30017352

[8] Lokmer A, Kuenzel S, Baines JF, Wegner KM. 2016 The role of tissue-specific microbiota in initial establishment success of Pacific oysters. Environ. Microbiol. **18**, 970-987. (10.1111/1462-2920.13163) 26695476

[9] Dupont S et al. 2020 Oyster hemolymph is a complex and dynamic ecosystem hosting bacteria, protists and viruses. Anim. Microbiome **2**, 1-16. (10.1186/s42523-020-00032-w) PMC7807429 33499958

[10] De Lorgeril J et al. 2018 Inefficient immune response is associated with microbial permissiveness in juvenile oysters affected by mass mortalities on field. Fish Shellfish Immunol. **77**, 156-163. (10.1016/j.fsi.2018.03.027) 29567138

[11] Pimentel ZT, Dufault-Thompson K, Russo KT, Scro AK, Smolowitz RM, Gomez-Chiarri M, Zhang Y. 2021 Microbiome analysis reveals diversity and function of *Mollicutes* associated with the Eastern oyster, *Crassostrea virginica*. mSphere **6**, e00227-21. (10.1128/mSphere.00227-21) 33980678 PMC8125052

[12] King WL, Siboni N, Kahlke T, Dove M, O'Connor W, Mahbub KR, Jenkins C, Seymour JR, Labbate M. 2020 Regional and oyster microenvironmental scale heterogeneity in the Pacific oyster bacterial community. FEMS Microbiol. Ecol. **96**, fiaa054. (10.1093/femsec/fiaa054) 32221598

[13] Unzueta-Martínez A, Scanes E, Parker LM, Ross PM, O'Connor W, Bowen JL. 2022 Microbiomes of the Sydney rock oyster are acquired through both vertical and horizontal transmission. Anim. Microbiome **4**, 32. (10.1186/s42523-022-00186-9) 35590396 PMC9118846

[14] Vezzulli L, Stagnaro L, Grande C, Tassistro G, Canesi L, Pruzzo C. 2018 Comparative 16SrDNA gene-based microbiota profiles of the Pacific oyster (*Crassostrea gigas*) and the Mediterranean mussel (*Mytilus galloprovincialis*) from a shellfish farm (Ligurian Sea, Italy). Microb. Ecol. **75**, 495-504. (10.1007/s00248-017-1051-6) 28803409

[15] Clerissi C, De Lorgeril J, Petton B, Lucasson A, Escoubas JM, Gueguen Y, Dégremont L, Mitta G, Toulza E. 2020 Microbiota composition and evenness predict survival rate of oysters confronted to Pacific oyster mortality syndrome. Front. Microbiol. **11**, 1-11. (10.3389/fmicb.2020.00311) 32174904 PMC7056673

[16] Offret C et al. 2023 Microbiota of the digestive glands and extrapallial fluids of clams evolve differently over time depending on the intertidal position. Microb. Ecol. **85**, 288-297. (10.1007/s00248-022-01959-0) 35066615

[17] Destoumieux-Garzón D, Canesi L, Oyanedel D, Travers MA, Charrière GM, Pruzzo C, Vezzulli L. 2020 Vibrio–bivalve interactions in health and disease. Environ. Microbiol. **22**, 4323-4341. (10.1111/1462-2920.15055) 32363732

[18] Duperron S, Gaudron SM, Rodrigues CF, Cunha MR, Decker C, Olu K. 2012 An overview of chemosynthetic symbioses in bivalves from the North Atlantic and Mediterranean Sea. Biogeosciences **10**, 3241-3267. (10.5194/bgd-9-16815-2012)

[19] Gros O, Elisabeth NH, Gustave SDD, Caro A, Dubilier N. 2012 Plasticity of symbiont acquisition throughout the life cycle of the shallow-water tropical lucinid *Codakia orbiculata* (Mollusca: Bivalvia): symbiont acquisition in lucinid clams. Environ. Microbiol. **14**, 1584-1595. (10.1111/j.1462-2920.2012.02748.x) 22672589

[20] Zhang G et al. 2012 The oyster genome reveals stress adaptation and complexity of shell formation. Nature **490**, 49-54. (10.1038/nature11413) 22992520

[21] Zhang L, Li L, Guo X, Litman GW, Dishaw LJ, Zhang G. 2015 Massive expansion and functional divergence of innate immune genes in a protostome. Sci. Rep. **5**, 8693. (10.1038/srep08693) 25732911 PMC4346834

[22] Paillard C, Gueguen Y, Wegner KM, Bass D, Pallavicini A, Vezzulli L, Arzul I. 2022 Recent advances in bivalve–microbiota interactions for disease prevention in aquaculture. Curr. Opin. Biotechnol. **73**, 225-232. (10.1016/j.copbio.2021.07.026) 34571318

[23] De Lorgeril J et al. 2018 Immune-suppression by OsHV-1 viral infection causes fatal bacteraemia in Pacific oysters. Nat. Commun. **9**, 4215. (10.1038/s41467-018-06659-3) 30310074 PMC6182001

[24] De San Nicolas N et al. 2022 Functional diversification of oyster big defensins generates antimicrobial specificity and synergy against members of the microbiota. Mar. Drugs **20**, 745. (10.3390/md20120745) 36547892 PMC9786018

[25] Fallet M et al. 2022 Early life microbial exposures shape the *Crassostrea gigas* immune system for lifelong and intergenerational disease protection. Microbiome **10**, 1-21. (10.1186/s40168-022-01280-5) 35659369 PMC9167547

[26] Clerissi C et al. 2023 A core of functional complementary bacteria infects oysters in Pacific oyster mortality syndrome. Anim. Microbiome **5**, 26. (10.1186/s42523-023-00246-8) 37138356 PMC10155333

[27] Kennedy VS, Newell RI, Eble AF. 1996. The eastern oyster *Crassostrea virginica*. College Park, MD: The Maryland Sea Grant College. https://repository.library.noaa.gov/view/noaa/45763.

[28] Brousseau DJ, Braun PC, Harper-Leatherman AS, Sullivan E, Baglivo JA. 2014 Antimicrobial activity in the pallial cavity fluids of the oyster *Crassostrea virginica* (Gmelin) from a highly impacted harbor in Western Long Island Sound. J. Shellfish Res. **33**, 719-725. (10.2983/035.033.0306)

[29] Espinosa EP, Koller A, Allam B. 2016 Proteomic characterization of mucosal secretions in the eastern oyster, *Crassostrea virginica*. J. Proteomics **132**, 63-76. (10.1016/j.jprot.2015.11.018) 26612663

[30] Garland CD, Nash GV, Mcmeekin TA. 1982 Absence of surface-associated microorganisms in adult oysters (*Crassostrea gigas*). Appl. Environ. Microbiol. **44**, 1205-1211. (10.1128/aem.44.5.1205-1211.1982) 7181503 PMC242169

[31] Braun PC, Brousseau DJ, Lecleir GR. 2019 Microbial inhibition by bacteria isolated from pallial cavity fluids and associated mucus of the Eastern oyster *Crassostrea virginica* (Gmelin). J. Shellfish Res. **38**, 565. (10.2983/035.038.0307)

[32] Desriac F, Le Chevalier P, Brillet B, Leguerinel I, Thuillier B, Paillard C, Fleury Y. 2014 Exploring the hologenome concept in marine bivalvia: haemolymph microbiota as a pertinent source of probiotics for aquaculture. FEMS Microbiol. Lett. **350**, 107-116. (10.1111/1574-6968.12308) 24286558

[33] Desriac F et al. 2020 Alterins produced by oyster-associated *Pseudoalteromonas* are antibacterial cyclolipopeptides with LPS-binding activity. Marine Drugs **18**, 630. (10.3390/md18120630) 33321943 PMC7764243

[34] Offret C et al. 2020 The marine intertidal zone shapes oyster and clam digestive bacterial microbiota. FEMS Microbiol. Ecol. **96**, fiaa078. (10.1093/femsec/fiaa078) 32353873

[35] Scanes E, Parker LM, Seymour JR, Siboni N, Dove MC, O'Connor WA, Ross PM. 2021 Microbiomes of an oyster are shaped by metabolism and environment. Sci. Rep. **11**, 21112. (10.1038/s41598-021-00590-2) 34702926 PMC8548560

[36] Scanes E, Parker LM, Seymour JR, Siboni N, King WL, Wegner KM, Dove MC, O'Connor WA, Ross PM. 2021 Microbiome response differs among selected lines of Sydney rock oysters to ocean warming and acidification. FEMS Microbiol. Ecol. **97**, fiab099. (10.1093/femsec/fiab099) 34190992

[37] Fernández N T, Mazón-Suástegui JM, Vázquez-Juárez R, Ascencio-Valle F, Romero J. 2014 Changes in the composition and diversity of the bacterial microbiota associated with oysters (*Crassostrea corteziensis*, *Crassostrea gigas* and *Crassostrea sikamea*) during commercial production. FEMS Microbiol. Ecol. **88**, 69-83. (10.1111/1574-6941.12270) 24325323

[38] Wegner KM, Volkenborn N, Peter H, Eiler A. 2013 Disturbance induced decoupling between host genetics and composition of the associated microbiome. BMC Microbiol. **13**, 1-12. (10.1186/1471-2180-13-252) 24206899 PMC3840651

[39] Hines IS, Markov Madanick J, Smith SA, Kuhn DD, Stevens AM. 2023 Analysis of the core bacterial community associated with consumer-ready Eastern oysters (*Crassostrea virginica*). PLoS ONE **18**, e0281747. (10.1371/journal.pone.0281747) 36812164 PMC9946220

[40] Lokmer A, Goedknegt MA, Thieltges DW, Fiorentino D, Kuenzel S, Baines JF, Wegner KM. 2016 Spatial and temporal dynamics of Pacific oyster hemolymph microbiota across multiple scales. Front. Microbiol. **7**, 1-18. (10.3389/fmicb.2016.01367) 27630625 PMC5006416

[41] Scanes E et al. 2021 Climate change alters the haemolymph microbiome of oysters. Mar. Pollut. Bull. **164**, 111991. (10.1016/j.marpolbul.2021.111991) 33485019

[42] Harwood CS, Canale-Parola E. 1984 Ecology of spirochetes. Ann. Rev. Microbiol. **38**, 161-192. (10.1146/annurev.mi.38.100184.001113) 6388490

[43] Schmitt P, Rosa RD, Duperthuy M, De Lorgeril J, Bachère E, Destoumieux-Garzón D. 2012 The antimicrobial defense of the Pacific oyster, *Crassostrea gigas*. How diversity may compensate for scarcity in the regulation of resident/pathogenic microflora. Front. Microbiol. **3**, 1-17. (10.3389/fmicb.2012.00160) 22783227 PMC3390580

[44] Escoubas J-M, Gourbal B, Duval D, Green TJ, Charrière GM, Destoumieux-Garzón D, Montagnani C. 2016 Immunity in Molluscs. In Encyclopedia of immunobiology: Academic P. Oxford: Elseviers Ltd.

[45] Rubio T et al. 2019 Species-specific mechanisms of cytotoxicity toward immune cells determine the successful outcome of Vibrio infections. Proc. Natl Acad. Sci. USA **116**, 201905747. (10.1073/pnas.1905747116) PMC6628822 31221761

[46] Oyanedel D et al. 2020 Vibrio splendidus O-antigen structure: a trade-off between virulence to oysters and resistance to grazers. Environ. Microbiol. **22**, 4264-4278. (10.1111/1462-2920.14996) 32219965

[47] Oyanedel D et al. 2023 Cooperation and cheating orchestrate *Vibrio* assemblages and polymicrobial synergy in oysters infected with OsHV-1 virus. Proc. Natl Acad. Sci. USA **120**, e2305195120. (10.1073/pnas.2305195120) 37751557 PMC10556616

[48] Zaidman-Rémy A et al. 2006 The *Drosophila* amidase PGRP-LB modulates the immune response to bacterial infection. Immunity **24**, 463-473. (10.1016/j.immuni.2006.02.012) 16618604

[49] Song X, Wang H, Xin L, Xu J, Jia Z, Wang L, Song L. 2016 The immunological capacity in the larvae of Pacific oyster *Crassostrea gigas*. Fish Shellfish Immunol. **49**, 461-469. (10.1016/j.fsi.2016.01.009) 26806166

[50] Tirapé A, Bacque C, Brizard R, Vandenbulcke F, Boulo V. 2007 Expression of immune-related genes in the oyster *Crassostrea gigas* during ontogenesis. Dev. Comp. Immunol. **31**, 859-873. (10.1016/j.dci.2007.01.005) 17337052

[51] Bachere E, Rosa RD, Schmitt P, Poirier AC, Merou N, Charriere GM, Destoumieux-Garzon D. 2015 The new insights into the oyster antimicrobial defense: cellular, molecular and genetic view. Fish Shellfish Immunol. **46**, 50-64. (10.1016/j.fsi.2015.02.040) 25753917

[52] Poirier AC, Schmitt P, Rosa RD, Vanhove AS, Kieffer-Jaquinod S, Rubio TP, Charriere GM, Destoumieux-Garzon D. 2014 Antimicrobial histones and DNA traps in invertebrate immunity: evidences in *Crassostrea gigas*. J. Biol. Chem. **289**, 24 821-24 831. (10.1074/jbc.M114.576546) PMC4155652 25037219

[53] Labreuche Y, Soudant P, Goncalves M, Lambert C, Nicolas J. 2006 Effects of extracellular products from the pathogenic *Vibrio aestuarianus* strain 01/32 on lethality and cellular immune responses of the oyster *Crassostrea gigas*. Dev. Comp. Immunol. **30**, 367-379. (10.1016/j.dci.2005.05.003) 16005965

[54] Oyanedel D, Rojas R, Brokordt K, Schmitt P. 2023 *Crassostrea gigas* oysters from a non-intensive farming area naturally harbor potentially pathogenic vibrio strains. J. Invertebr. Pathol. **196**, 107856. (10.1016/j.jip.2022.107856) 36414122

[55] Destoumieux-Garzón D, Rosa RD, Schmitt P, Barreto C, Vidal-Dupiol J, Mitta G, Gueguen Y, Bachère E. 2016 Antimicrobial peptides in marine invertebrate health and disease. Phil. Trans. R. Soc. B **371**, 20150300. (10.1098/rstb.2015.0300) 27160602 PMC4874397

[56] Gerdol M, Schmitt P, Venier P, Rocha G, Rosa RD, Destoumieux-Garzón D. 2020 Functional insights from the evolutionary diversification of big defensins. Front. Immunol. **11**, 1-16. (10.3389/fimmu.2020.00758) 32425943 PMC7203481

[57] Schmitt P, Wilmes M, Pugniere M, Bachere E, Sahl H, Schneider T, Destoumieux-Garzon D. 2010 Insight into invertebrate defensin mechanism of action. J. Biol. Chem. **285**, 29 208-29 216. (10.1074/jbc.M110.143388) PMC2937951 20605792

[58] Gonzalez M et al. 2007 Evidence of a bactericidal permeability increasing protein in an invertebrate, the *Crassostrea gigas* Cg-BPI. Proc. Natl Acad. Sci. USA **104**, 17 759-17 764. (10.1073/pnas.0702281104) PMC2077063 17965238

[59] Loth K et al. 2019 The ancestral N-terminal domain of big defensins drives bacterially triggered assembly into antimicrobial nanonets karine. mBio **10**, e01821-19. (10.1128/mBio.01821-19) 31641083 PMC6805989

[60] Mitta G, Vandenbulcke F, Noel T, Romestand B, Beauvillain JC, Salzet M, Roch P. 2000 Differential distribution and defence involvement of antimicrobial peptides in mussel. J. Cell Sci. **113**, 2759-2769. (10.1242/jcs.113.15.2759) 10893191

[61] Schmitt P, Gueguen Y, Desmarais E, Bachère E, de Lorgeril J. 2010 Molecular diversity of antimicrobial effectors in the oyster *Crassostrea gigas*. BMC Evol. Biol. **10**, 23. (10.1186/1471-2148-10-23) 20100329 PMC2823732

[62] Schmitt P, de Lorgeril J, Gueguen Y, Destoumieux-Garzón D, Bachère E. 2012 Expression, tissue localization and synergy of antimicrobial peptides and proteins in the immune response of the oyster *Crassostrea gigas*. Dev. Comp. Immunol. **37**, 363-370. (10.1016/j.dci.2012.01.004) 22327168

[63] González R, Gonçalves AT, Rojas R, Brokordt K, Rosa RD, Schmitt P. 2020 Host defense effectors expressed by hemocytes shape the bacterial microbiota from the scallop hemolymph. Front. Immunol. **11**, 1-13. (10.3389/fimmu.2020.599625) 33281827 PMC7689009

[64] Hanson MA, Dostálová A, Ceroni C, Poidevin M, Kondo S, Lemaître B. 2019 Synergy and remarkable specificity of antimicrobial peptides *in vivo* using a systematic knockout approach (eLife (2019) 8 PII: e48778). eLife **8**, 1-24. (10.7554/eLife.48778) PMC6398976 30803481

[65] Hanson MA, Grollmus L, Lemaitre B. 2022 Ecology-relevant bacteria drive the evolution of host antimicrobial peptides in *Drosophila*. Science **381**, eadg5725. (10.1101/2022.12.23.521774) 37471548

[66] Rosa RD, Alonso P, Santini A, Vergnes A, Bachère E. 2015 High polymorphism in big defensin gene expression reveals presence-absence gene variability (PAV) in the oyster *Crassostrea gigas*. Dev. Comp. Immunol. **49**, 231-238. (10.1016/j.dci.2014.12.002) 25482648

[67] Wongpanya R, Sengprasert P, Amparyup P, Tassanakajon A. 2017 A novel C-type lectin in the black tiger shrimp *Penaeus monodon* functions as a pattern recognition receptor by binding and causing bacterial agglutination. Fish Shellfish Immunol. **60**, 103-113. (10.1016/j.fsi.2016.11.042) 27876622

[68] Wang X-W, Xu J-D, Zhao X-F, Vasta GR, Wang J-X. 2014 A shrimp C-type lectin inhibits proliferation of the hemolymph microbiota by maintaining the expression of antimicrobial peptides. J. Biol. Chem. **289**, 11 779-11 790. (10.1074/jbc.M114.552307) PMC4002086 24619414

[69] Zhang Y-X, Zhang M-L, Wang X-W. 2021 C-type lectin maintains the homeostasis of intestinal microbiota and mediates biofilm formation by intestinal bacteria in shrimp. J. Immunol. **206**, 1140-1150. (10.4049/jimmunol.2000116) 33526439

[70] Song X et al. 2019 A single-CRD C-type lectin (CgCLec-3) with novel DIN motif exhibits versatile immune functions in *Crassostrea gigas*. Fish Shellfish Immunol. **92**, 772-781. (10.1016/j.fsi.2019.07.001) 31279080

[71] Mao F et al. 2020 Opsonic character of the plasma proteins in phagocytosis-dependent host response to bacterial infection in a marine invertebrate, *Crassostrea gigas*. Dev. Comp. Immunol. **106**, 103596. (10.1016/j.dci.2019.103596) 31877328

[72] Moné Y, Gourbal B, Duval D, Du Pasquier L, Kieffer-Jaquinod S, Mitta G. 2010 A large repertoire of parasite epitopes matched by a large repertoire of host immune receptors in an invertebrate host/parasite model. PLoS Negl. Trop. Dis. **4**, e813. (10.1371/journal.pntd.0000813) 20838648 PMC2935394

[73] Portet A, Galinier R, Pinaud S, Portela J, Nowacki F, Gourbal B, Duval D. 2018 BgTEP: an antiprotease involved in innate immune sensing in *Biomphalaria glabrata*. Front. Immunol. **9**, 1206. (10.3389/fimmu.2018.01206) 29899746 PMC5989330

[74] Levashina EA, Moita LF, Blandin S, Vriend G, Lagueux M, Kafatos FC. 2001 Conserved role of a complement-like protein in phagocytosis revealed by dsRNA knockout in cultured cells of the mosquito, *Anopheles gambiae*. Cell **104**, 709-718. (10.1016/S0092-8674(01)00267-7) 11257225

[75] Stroschein-Stevenson SL, Foley E, O'Farrell PH, Johnson AD. 2005 Identification of *Drosophila* gene products required for phagocytosis of *Candida albicans*. PLoS Biol. **4**, e4. (10.1371/journal.pbio.0040004) 16336044 PMC1310651

[76] Rosenstiel P et al. 2007 Regulation of *DMBT1* via NOD2 and TLR4 in intestinal epithelial cells modulates bacterial recognition and invasion. J. Immunol. **178**, 8203-8211. (10.4049/jimmunol.178.12.8203) 17548659

[77] Arezes J. 2015 Hepcidin-induced hypoferremia is a critical host defense mechanism against the siderophilic bacterium *Vibrio vulnificus*. Cell Host Microbe **17**, 47-57. (10.1038/jid.2014.371) 25590758 PMC4296238

[78] Mcfall-Ngai M et al. 2013 Animals in a bacterial world, a new imperative for the life sciences. Proc. Natl Acad. Sci. USA **110**, 3229-3236. (10.1073/pnas.1218525110) 23391737 PMC3587249

[79] Jarchum I, Pamer EG. 2011 Regulation of innate and adaptive immunity by the commensal microbiota. Curr. Opin. Immunol. **23**, 353-360. (10.1016/j.coi.2011.03.001) 21466955 PMC3109238

[80] Round JL, Mazmanian SK. 2009 The gut microbiota shapes intestinal immune responses during health and disease. Nat. Rev. Immunol. **9**, 313-323. (10.1038/nri2515) 19343057 PMC4095778

[81] Ansaldo E, Farley TK, Belkaid Y. 2021 Control of immunity by the microbiota. Annu. Rev. Immunol. **39**, 449-479. (10.1146/annurev-immunol-093019-112348) 33902310

[82] Carlson JS, Short SM, Angleró-Rodríguez YI, Dimopoulos G. 2020 Larval exposure to bacteria modulates arbovirus infection and immune gene expression in adult *Aedes aegypti*. Dev. Comp. Immunol. **104**, 103540. (10.1016/j.dci.2019.103540) 31726064

[83] Galindo-Villegas J, García-Moreno D, De Oliveira S, Meseguer J, Mulero V. 2012 Regulation of immunity and disease resistance by commensal microbes and chromatin modifications during zebrafish development. Proc. Natl Acad. Sci. USA **109**, E2605-E2614. (10.1073/pnas.1209920109) 22949679 PMC3465450

[84] Gensollen T, Iyer SS, Kasper DL, Blumberg RS. 2016 How colonization by microbiota in early life shapes the immune system. Science **352**, 539-544. (10.1126/science.aad9378) 27126036 PMC5050524

[85] Kelly C, Salinas I. 2017 Under pressure: interactions between commensal microbiota and the teleost immune system. Front. Immunol. **8**, 559. (10.3389/fimmu.2017.00559) 28555138 PMC5430139

[86] Rader B, McAnulty SJ, Nyholm SV. 2019 Persistent symbiont colonization leads to a maturation of hemocyte response in the *Euprymna scolopes*/*Vibrio fischeri* symbiosis. MicrobiologyOpen **8**, e858. (10.1002/mbo3.858) 31197972 PMC6813443

[87] Weiss BL, Wang J, Aksoy S. 2011 Tsetse immune system maturation requires the presence of obligate symbionts in larvae. PLoS Biol. **9**, e1000619. (10.1371/journal.pbio.1000619) 21655301 PMC3104962

[88] Arrieta M-C, Stiemsma LT, Amenyogbe N, Brown EM, Finlay B. 2014 The intestinal microbiome in early life: health and disease. Front. Immunol. **5**, 427. (10.3389/fimmu.2014.00427) 25250028 PMC4155789

[89] Fanucchi S, Domínguez-Andrés J, Joosten LAB, Netea MG, Mhlanga MM. 2021 The intersection of epigenetics and metabolism in trained immunity. Immunity **54**, 32-43. (10.1016/j.immuni.2020.10.011) 33220235

[90] Lindermayr C, Rudolf EE, Durner J, Groth M. 2020 Interactions between metabolism and chromatin in plant models. Mol. Metab. **38**, 100951. (10.1016/j.molmet.2020.01.015) 32199818 PMC7300381

[91] Gawra J et al. 2023 Epigenetic variations are more substantial than genetic variations in rapid adaptation of oyster to Pacific oyster mortality syndrome. Sci. Adv. **9**, eadh8990. (10.1126/sciadv.adh8990) 37683000 PMC10491289

[92] Aristizabal MJ et al. 2020 Biological embedding of experience: a primer on epigenetics. Proc. Natl Acad. Sci. USA **117**, 23 261-23 269. (10.1073/pnas.1820838116) 31624126 PMC7519272

[93] Turner BM. 2009 Epigenetic responses to environmental change and their evolutionary implications. Phil. Trans. R. Soc. B **364**, 3403-3418. (10.1098/rstb.2009.0125) 19833651 PMC2781845

[94] Yin J, Zhou M, Lin Z, Li QQ, Zhang Y-Y. 2019 Transgenerational effects benefit offspring across diverse environments: a meta-analysis in plants and animals. Ecol. Lett. **22**, 1976-1986. (10.1111/ele.13373) 31436014

[95] McCoy KD, Burkhard R, Geuking MB. 2019 The microbiome and immune memory formation. Immunol. Cell Biol. **97**, 625-635. (10.1111/imcb.12273) 31127637

[96] Abt MC et al. 2012 Commensal bacteria calibrate the activation threshold of innate antiviral immunity. Immunity **37**, 158-170. (10.1016/j.immuni.2012.04.011) 22705104 PMC3679670

[97] Erttmann SF, Swacha P, Aung KM, Brindefalk B, Jiang H, Härtlova A, Uhlin BE, Wai SN, Gekara NO. 2022 The gut microbiota prime systemic antiviral immunity via the cGAS-STING-IFN-I axis. Immunity **55**, 847-861. (10.1016/j.immuni.2022.04.006) 35545033

[98] Hoang KL, King KC. 2022 Symbiont-mediated immune priming in animals through an evolutionary lens. Microbiology **168**, 001181. (10.1099/mic.0.001181) 35442184

[99] Holt CC, Bass D, Stentiford GD, Van Der Giezen M. 2021 Understanding the role of the shrimp gut microbiome in health and disease. J. Invertebr. Pathol. **186**, 107387. (10.1016/j.jip.2020.107387) 32330478

[100] Huang Z et al. 2020 Microecological Koch's postulates reveal that intestinal microbiota dysbiosis contributes to shrimp white feces syndrome. Microbiome **8**, 32. (10.1186/s40168-020-00802-3) 32156316 PMC7065354

[101] Ichinohe T, Pang IK, Kumamoto Y, Peaper DR, Ho JH, Murray TS, Iwasaki A. 2011 Microbiota regulates immune defense against respiratory tract influenza A virus infection. Proc. Natl Acad. Sci. USA **108**, 5354-5359. (10.1073/pnas.1019378108) 21402903 PMC3069176

[102] Näpflin K, Schmid-Hempel P. 2016 Immune response and gut microbial community structure in bumblebees after microbiota transplants. Proc. R. Soc. B. **283**, 20160312. (10.1098/rspb.2016.0312) PMC4892793 27226466

[103] Sansone CL, Cohen J, Yasunaga A, Xu J, Osborn G, Subramanian H, Gold B, Buchon N, Cherry S. 2015 Microbiota-dependent priming of antiviral intestinal immunity in *Drosophila*. Cell Host Microbe **18**, 571-581. (10.1016/j.chom.2015.10.010) 26567510 PMC4648705

[104] Prigot-Maurice C, Beltran-Bech S, Braquart-Varnier C. 2022 Why and how do protective symbionts impact immune priming with pathogens in invertebrates? Dev. Comp. Immunol. **126**, 104245. (10.1016/j.dci.2021.104245) 34453995

[105] Lafont M et al. 2020 A sustained immune response supports long-term antiviral immune priming in the Pacific oyster, *Crassostrea gigas*. mBio **11**, 1-17. (10.1128/mBio.02777-19) PMC7064767 32156821

[106] Wang W, Wang L, Liu Z, Song X, Yi Q, Yang C, Song L. 2020 The involvement of TLR signaling and anti-bacterial effectors in enhanced immune protection of oysters after *Vibrio splendidus* pre-exposure. Dev. Comp. Immunol. **103**, 103498. (10.1016/j.dci.2019.103498) 31525382

[107] Bourne DG, Morrow KM, Webster NS. 2016 Insights into the coral microbiome: underpinning the health and resilience of reef ecosystems. Annu. Rev. Microbiol. **70**, 317-340. (10.1146/annurev-micro-102215-095440) 27482741

[108] Baldassarre L, Ying H, Reitzel AM, Franzenburg S, Fraune S. 2022 Microbiota mediated plasticity promotes thermal adaptation in the sea anemone *Nematostella vectensis*. Nat. Commun. **13**, 3804. (10.1038/s41467-022-31350-z) 35778405 PMC9249911

[109] Voolstra CR, Ziegler M. 2020 Adapting with microbial help: microbiome flexibility facilitates rapid responses to environmental change. Bioessays **42**, 2000004. (10.1002/bies.202000004) 32548850

[110] Hou K et al. 2022 Microbiota in health and diseases. Signal Transduct. Target Ther. **7**, 135. (10.1038/s41392-022-00974-4) 35461318 PMC9034083

[111] Jones HR, Johnson KM, Kelly MW. 2019 Synergistic effects of temperature and salinity on the gene expression and physiology of *Crassostrea virginica*. Integr. Comp. Biol. **59**, 306-319. (10.1093/icb/icz035) 31076748

[112] King WL, Jenkins C, Seymour JR, Labbate M. 2019 Oyster disease in a changing environment: decrypting the link between pathogen, microbiome and environment. Mar. Environ. Res. **143**, 124-140. (10.1016/j.marenvres.2018.11.007) 30482397

[113] Hemraj DA, Falkenberg LJ, Cheung K, Man L, Carini A, Russell BD. 2023 Acidification and hypoxia drive physiological trade-offs in oysters and partial loss of nutrient cycling capacity in oyster holobiont. Front. Ecol. Evol. **11**, 1083315. (10.3389/fevo.2023.1083315)

[114] Lokmer A, Wegner KM. 2015 Hemolymph microbiome of Pacific oysters in response to temperature, temperature stress and infection. ISME J. **9**, 670-682. (10.1038/ismej.2014.160) 25180968 PMC4331581

[115] Green TJ, Siboni N, King WL, Labbate M, Seymour JR, Raftos D. 2019 Simulated marine heat wave alters abundance and structure of vibrio populations associated with the Pacific oyster resulting in a mass mortality event. Microb. Ecol. **77**, 736-747. (10.1007/s00248-018-1242-9) 30097682

[116] Lupo C, Dutta B, Petton S, Ezanno P, Tourbiez D, Travers M, Pernet F, Bacher C. 2020 Spatial epidemiological modelling of infection by *Vibrio aestuarianus* shows that connectivity and temperature control oyster mortality. Aquacult. Environ. Interact. **12**, 511-527. (10.3354/aei00379)

[117] Lasa A et al. 2019 Dynamics of the Pacific oyster pathobiota during mortality episodes in Europe assessed by 16S rRNA gene profiling and a new target enrichment next-generation sequencing strategy. Environ. Microbiol. **21**, 4548-4562. (10.1111/1462-2920.14750) 31325353 PMC7379488

[118] Parizadeh L, Tourbiez D, Garcia C, Haffner P, Dégremont L, Le Roux F, Travers MA. 2018 Ecologically realistic model of infection for exploring the host damage caused by *Vibrio aestuarianus*. Environ. Microbiol. **20**, 4343-4355. (10.1111/1462-2920.14350) 29974612

[119] Petton B, Destoumieux-Garzon D, Pernet F, Toulza E, De Lorgeril J, Degremont L, Mitta G. 2021 The Pacific oyster mortality syndrome, a polymicrobial and multifactorial disease: state of knowledge and future directions. Front. Immunol. **12**, 1-10. (10.3389/fimmu.2021.630343) PMC7930376 33679773

[120] Petton B, Bruto M, James A, Labreuche Y, Alunno-Bruscia M, Le Roux F. 2015 *Crassostrea gigas* mortality in France: the usual suspect, a herpes virus, may not be the killer in this polymicrobial opportunistic disease. Front. Microbiol. **6**, 1-10. (10.3389/fmicb.2015.00686) 26217318 PMC4491618

[121] Food and Agricultural Organization of the United Nations. 2016 Probiotics in animal nutrition: production, impact and regulation. Rome, Italy: Food and Agricultural Organization of the United Nations.

[122] Getachew T. 2016 A review on effects of probiotic supplementation in poultry performance and cholesterol levels of egg and meat. https://www.academia.edu/30610184/A_Review_on_Effects_of_Probiotic_Supplementation_in_Poultry_Performance_and_Cholesterol_Levels_of_Egg_and_Meat.

[123] Khademzade O, Zakeri M, Haghi M, Mousavi SM. 2020 The effects of water additive *Bacillus cereus* and *Pediococcus acidilactici* on water quality, growth performances, economic benefits, immunohematology and bacterial flora of whiteleg shrimp *(Penaeus vannamei* Boone, 1931) reared in earthen ponds. Aquat. Res. **51**, 1759-1770. (10.1111/are.14525)

[124] Yeh H, Skubel SA, Patel H, Cai Shi D, Bushek D, Chikindas ML. 2020 From farm to fingers: an exploration of probiotics for oysters, from production to human consumption. Probiotics Antimicrobial Proteins **12**, 351-364. (10.1007/s12602-019-09629-3) 32056150

[125] Madison D, Schubiger C, Spencer L, Mueller RS, Lagdon C. 2022 A marine probiotic treatment against the bacterial pathogen *Vibrio coralliilyticus* to improve the performance of Pacific (*Crassostrea gigas*) and Kumamoto (*C. sikamea*) oyster larvae. *bioRxiv* 491202. (10.1101/2022.05.09.491202)

[126] García-Bernal M, Medina-Marrero R, Campa-Córdova ÁI, Mazón-Suástegui JM. 2019 Growth and antioxidant response of juvenile oysters *Crassostrea sikamea* and *Crassostrea corteziensis* treated with *Streptomyces* strains. Arq. Bras. Med. Vet. Zootec. **71**, 1993-1998. (10.1590/1678-4162-11225)

[127] Dugeny E, Lorgeril J, Petton B, Toulza E, Gueguen Y, Pernet F. 2022 Seaweeds influence oyster microbiota and disease susceptibility. J. Anim. Ecol. **91**, 805-818. (10.1111/1365-2656.13662) 35137405

[128] De Schryver P, Defoirdt T, Sorgeloos P. 2014 Early mortality syndrome outbreaks: a microbial management issue in shrimp farming? PLoS Pathog. **10**, e1003919. (10.1371/journal.ppat.1003919) 24763380 PMC3999206

[129] De Schryver P, Vadstein O. 2014 Ecological theory as a foundation to control pathogenic invasion in aquaculture. ISME J. **8**, 2360-2368. (10.1038/ismej.2014.84) 24892581 PMC4260705

[130] Bossier P et al. 2016 Microbial community management in aquaculture. Procedia Food Sci. **6**, 37-39. (10.1016/j.profoo.2016.02.007)

[131] Vadstein O, Attramadal KJK, Bakke I, Olsen Y. 2018 K-selection as microbial community management strategy: a method for improved viability of larvae in aquaculture. Front. Microbiol. **9**, 2730. (10.3389/fmicb.2018.02730) 30487782 PMC6246659

[132] Kumar V, Roy S, Behera BK, Swain HS, Das BK. 2021 Biofloc microbiome with bioremediation and health benefits. Front. Microbiol. **12**, 741164. (10.3389/fmicb.2021.741164) 34912305 PMC8667556

[133] Gourbal B, Pinaud S, Beckers GJM, Van Der Meer JWM, Conrath U, Netea MG. 2018 Innate immune memory: an evolutionary perspective. Immunol. Rev. **283**, 21-40. (10.1111/imr.12647) 29664574

[134] Lafont M, Goncalves P, Guo X, Montagnani C, Raftos D, Green T. 2019 Transgenerational plasticity and antiviral immunity in the Pacific oyster (*Crassostrea gigas*) against Ostreid herpesvirus 1 (OsHV-1). Dev. Comp. Immunol. **91**, 17-25. (10.1016/j.dci.2018.09.022) 30278186

[135] Yang W et al. 2021 Immune priming in shellfish: a review and an updating mechanistic insight focused on cellular and humoral responses. Aquaculture **530**, 735831. (10.1016/j.aquaculture.2020.735831)

[136] Stevick RJ, Post AF, Gómez-Chiarri M. 2021 Functional plasticity in oyster gut microbiomes along a eutrophication gradient in an urbanized estuary. Anim. Microbiome **3**, 5. (10.1186/s42523-020-00066-0) 33499983 PMC7934548

[137] Piel D et al. 2022 Phage–host coevolution in natural populations. Nat. Microbiol. **7**, 1075-1086. (10.1038/s41564-022-01157-1) 35760840

[138] Breitbart M, Bonnain C, Malki K, Sawaya NA. 2018 Phage puppet masters of the marine microbial realm. Nat. Microbiol. **3**, 754-766. (10.1038/s41564-018-0166-y) 29867096

[139] Azema P, Travers MA, Benabdelmouna A, Degremont L. 2016 Single or dual experimental infections with *Vibrio aestuarianus* and OsHV-1 in diploid and triploid *Crassostrea gigas* at the spat, juvenile and adult stages. J. Invertebr. Pathol. **139**, 92-101. (10.1016/j.jip.2016.08.002) 27503207

